# Utilization of Waste Date Palm Leaves Biomass Ensiled with Malic or Lactic Acids in Diets of Farafra Ewes under Tropical Conditions

**DOI:** 10.3390/ani12111432

**Published:** 2022-06-01

**Authors:** Gouda A. Gouda, Ahmed E. Kholif, Hatem A. Hamdon, Ayman Y. Kassab, Amlan K. Patra

**Affiliations:** 1 Dairy Science Department, National Research Centre, 33 Bohouth St. Dokki, Giza 12622, Egypt; gagouda@gmail.com; 2 Department of Animal Production, Faculty of Agriculture, New Valley University, New Valley 72511, Egypt; hamdon@nv.aun.edu.eg (H.A.H.); ayman15@yahoo.com (A.Y.K.); 3 Department of Animal Nutrition, West Bengal University of Animal and Fishery Sciences, 37 K.B. Sarani, Kolkata 700037, India; patra_amlan@yahoo.com

**Keywords:** date palm leaves, ensiling, milk fatty acid, milk production, organic acid, ruminal fermentation

## Abstract

**Simple Summary:**

In many places and under certain conditions, feeding shrubs and trees such as date palm leaves to goats and sheep is a common practice to reduce feed costs. The main problem with such materials is the low nutritive value. Therefore, improving their nutritive value before feeding can overcome the problem of low nutritive value. Ensiling with organic acids can be used as a good strategy to increase the digestion and intake of date palm leaves and other agricultural byproducts. The inclusion of malic or lactic acid during ensiling can improve the quality of silages, resulting in improved performance (daily gain or milk production) when these materials are fed to ruminants, resulting in increased profitability. This is the first study to evaluate date palm leaves ensiled with malic or lactic acids in the diet of lactating ewes under desert conditions.

**Abstract:**

The aim of the current study was to evaluate the ensiling of date palm leaves (DPL) with organic acids (lactic or malic acid) for 45 day as a feed for lactating ewes under desert conditions. Two weeks before expected parturition, 50 multiparous lactating Farafra ewes (mean ± SD: 2 ± 0.3 parity, 34 ± 1.9 kg bodyweight, 25 ± 2.4 months of age, and 555 ± 13.0 g/day of previous milk production) were equally divided into five treatments in a completely randomized design for 90 day. The ewes in the control treatment were offered a diet composed of a concentrate feed mixture and DPL at 60:40 on a dry matter (DM) basis ensiled without additive. In the other treatments, DPL (ensiled without organic acids) in the control treatment was replaced with DPL ensiled with lactic or malic acid (at 5 g/kg DM) at 50 or 100% levels. Organic acids linearly and quadratically increased (*p* < 0.01) DPL and total intakes and digestibilities of DM, organic matter, crude protein, and nonstructural carbohydrates without affecting fiber digestibility. Malic and lactic acid treatment also increased the concentrations of ruminal total volatile fatty acids, acetate, propionate, and ammonia-N. Additionally, malic and lactic acid-treated DPL increased serum glucose concentration and total antioxidant capacity. Without affecting daily actual milk production, treatments increased (*p* < 0.001) the daily production of energy-corrected milk (ECM), fat-corrected milk (FCM), milk energy output, milk contents of fats, and feed efficiency. Organic acid-treated DPL increased (*p* < 0.05) the proportions of total polyunsaturated fatty acids and total conjugated linoleic acids and the unsaturated to saturated fatty acid ratio in milk. It is concluded that feeding DPL ensiled with malic or lactic acid at 20 or 40% of total diet DM increased daily ECM and FCM production, nutrient utilization efficiency, and milk quality. No differences were observed between lactic and malic acid treatment of DPL during ensiling; therefore, both of them are recommended to treat DPL for silage preparation.

## 1. Introduction

One of the main problems faced by livestock farmers in semi-arid and arid regions is the shortage of available feed ingredients. Therefore, exploring alternative feeds (e.g., agricultural byproducts) and improving the nutritive value of available ones are recommended. Mechanical, chemical, and biological treatments of low-quality feeds can be used for this purpose [1,2]. Date palm (*Phoenix dactilifera*) leaves (DPL) byproduct is one of the major crop residues in Egypt and many other arid regions. In Egypt, approximately 650,000 tons of leaves’ dry matter (DM) is produced annually from about 14 million trees [3,4]. DPL are not properly utilized due to high fiber (43–73% neutral detergent fiber (NDF) [5]) and low crude protein (CP) (4.2 to 16.5% of DM [6,7]) contents and low nutritive value and digestibility. Ensiling low-quality forages with silage additives is common to improve their fermentation profile and nutritive value and to reduce adverse bacterial growth and negative effects of some antinutritional factors in feeds [8]. However, ensiling is not effective with some feeds such as DPL due to their strong physical structure and low water-soluble carbohydrate content (<1%) [9], resulting in difficulty in making quality silage by natural fermentation. Therefore, some additives should be used to improve the ensiling process to improve the fermentation of silage. Organic acids include lactic acid, fumaric acid, malic acid, and formic acid, with good abilities to improve feed utilization and nutritive value of feeds [10]. Organic acids also have been applied to improve silage fermentation characteristics of many unconventional feeds [11,12]. The application of organic acids in silage (e.g., alfalfa) has lowered pH and proteolysis, improved fermentation quality, and retarded the growth of undesirable spoilage bacteria such as aerobes and enterobacteria [13]. This ensures a favorable environment for the rapid growth of lactic acid-producing bacteria in silages. Moreover, with different modes of action between malic and lactic acid due to the difference in chemical composition, nature, and metabolism by microbes during ensiling, organic acids stabilize ruminal pH, stimulate ruminal bacterial growth and the activity of some ruminal species (e.g., *Selenomonas ruminantium*), and enhance ruminant performance [10,14]. Additionally, they have been documented to reduce ruminal lactate production and may stimulate ruminal lactate-utilizing bacteria, e.g., *Megasphaera elsdenii* [15], and increase the production of ruminal volatile fatty acids (VFA) and propionate [12], nutrient digestibility, and milk production [10]. Moreover, Yang et al. [16] observed that steeping corn in tap water containing 1% lactic acid (wt/vol) for 48 h modulated the metabolic profiles of the rumen and increased ruminal pH in steers fed a high-concentrate diet.

However, the effect of feeding malic acid- or lactic acid-treated DPL on the production performance of ruminants is lacking. We hypothesized that ensiling DPL with lactic or malic acid might improve their quality as a feed for lactating ewes. In addition, we hypothesized that supplementation with organic acids might be an effective way of increasing ruminal pH, resulting in improved ruminal digestion and fermentation of feeds. Therefore, the aim of the current study was to evaluate the feeding of DPL ensiled with lactic or malic acid in the diet of lactating Farafra ewes on feed intake, digestion, milk production and composition, and ruminal fermentation under desert conditions.

## 2. Materials and Methods

### 2.1. Study Location

This lactational experiment was carried out in the experimental farm of the Department of Animal Production, Faculty of Agriculture of New Valley, Al Kharga City (25°26′ N and 30°32′ E). The maximum and minimum mean temperatures during the study were 39.5 ± 1.84 °C and 32.0 ± 1.37 °C, respectively. The temperature humidity index (THI) during the experimental period fluctuated between 77.0 and 84.7 calculated using the equation: THI = (0.8 × air temperature) + ((%relative humidity/100) × (air temperature−14.4)) + 46.4 [17]. The chemical analyses of samples were performed at the laboratory of Dairy Animal Production, National Research Centre, Egypt.

### 2.2. Date Palm Leaves

Fresh DPL were collected from different sites in the New Valley Governorate (Egypt) and sundried for 10 days [4]. Thereafter, DPL were ensiled under anaerobic conditions for 45 days using tightly closed polythene bag silos. Briefly, the chopped DPL were spread with a urea–molasses solution containing urea and molasses each at a 4% level. Before ensiling, the moisture content (initial moisture of 100 g/kg fresh matter) was increased to reach about 350 to 400 g/kg using the urea–molasses solution. Three types of DPL were prepared: DPL ensiled without additives, and DPL ensiled with malic acid or lactic acid. Malic and lactic acids (Sigma-Aldrich, Steinheim, Germany) were individually spread over during ensiling at 5 g/kg DM. Afterward, individual materials (i.e., DPL and organic acids) were mixed for homogenization. The materials were packed into 50 polythene bag silos (40 × 70 cm) for each silage type and compressed manually to create a semi-anaerobic environment. The quality of ensiled DPL (i.e., DPL ensiled without or with organic acids) after ensiling and before feeding was assessed using a sample (200 g fresh weight randomly collected from five bags) mixed with 800 mL distilled water and homogenized for 3 min with a blender and filtrated through four-layer cheesecloth. The filtrate was collected for measurement of pH using a digital pH meter, ammonia-N (NH_3_-N) according to AOAC [18], and volatile fatty acids (VFA) according to AOAC [18]. The aflatoxin (AF_1_) concentration was measured in silage with the use of a Fluorometer, Series-4 (Vicam, Milford, MA, USA), based on the methods described by AOAC [18].

### 2.3. Ewes and Management

Two weeks before expected parturition, 50 lactating Farafra ewes (mean ± SD: 2 ± 0.3 parity, 34 ± 1.9 kg bodyweight, 25 ± 2.4 months of age, and 555 ± 13.0 g/d of previous milk production) were assigned randomly to five treatments (*n* = 10 ewes/treatment) in a completely randomized design. The experiment was continued for 90 day. Ewes were housed individually in semi-opened concrete floor pens (1.5 m^2^/sheep). Each pen contained a feeder and a bucket for supplying fresh water ad libitum. Sheep were offered a diet comprising (per kg DM) 600 g of concentrate feed mixture and 400 g of ensiled DPL plus 10% extra allowance for the DPL. In the control group, DPL ensiled without organic acids was fed to ewes. In the treatment groups, ensiled DPL in the control group was replaced with DPL that was ensiled with malic (MAL50 and MAL100, respectively) or lactic acid (LAC50 and LAC100, respectively) at 50 or 100% levels. The concentrate feed mixture consisted of (per kg DM): 540 g corn, 250 g wheat bran, 180 g soybean meal, 20 g limestone, 5 g sodium chloride, and 5 g minerals and vitamins mixture. Ewes were offered the required fixed amount (510 g DM/d/ewe in all groups) of concentrate feed mixture, followed by the ensiled DPL. Diets were prepared to meet the nutrient requirements of lactating ewes according to NRC [19] recommendations. The ewes consumed the offered amount of concentrate mixture. Adjustments for DPL intake were made to the quantity of the diet offered to ensure the collection of 10% orts for DPL. The ingredient and chemical compositions of the experimental diets are presented in Table 1. Ewes were individually weighed monthly on a digital multipurpose platform scale. Diets were randomly sampled daily and stored pending chemical analyses after drying at 60 °C in a forced-air oven for 48 h [18].

### 2.4. Feed Intake and Nutrient Apparent Digestibility

Three digestibility trials were conducted during the last 10 day of each month (day 20–30, day 50–60, and day 80–90). On each day, the offered feeds and orts amounts were recorded. Acid insoluble ash was used as an internal indigestibility marker. Ferret et al. [20] equations were used to determine the apparent digestion coefficients of nutrients. During sample collection periods, daily feed intakes of concentrate and DPL were measured (feed offered–orts from the previous day’s feeding). Daily orts were individually collected and pooled for each ewe before sampling. During collection periods, individual fecal samples from all ewes were collected twice daily at 07:00 and 15:00 h, dried at 60 °C in a forced-air oven for 48 h, and pooled per ewe.

Composited samples of dried feed, orts, and feces were ground to pass through a 1-mm screen using a Wiley mill and analyzed for DM, ash, nitrogen, and ether extract (EE) according to AOAC [18] official methods. Neutral detergent fiber with the use of alpha-amylase and sodium sulfite and expressed exclusive of residual ash [21] and acid detergent fiber (ADF) expressed exclusive of residual ash [18] and lignin by solubilization of cellulose in the ADF residue with sulfuric acid [21] were analyzed. Concentrations (g/kg DM) of nonstructural carbohydrates (NSC), cellulose, hemicellulose, and organic matter (OM) were calculated.

### 2.5. Sampling and Analysis of Rumen Fluid

On the last day of each month period, ruminal fluid was collected in the morning at 3 h postfeeding using a stomach tube into a volumetric flask. About 100 mL of ruminal fluid was collected from all ewes of each treatment; the first 50 mL of the rumen fluid samples were discarded to avoid saliva contamination, and the rumen contents were strained through four layers of cheesecloth. Ammonia-N analysis was performed using a subsample of 5 mL of strained ruminal fluid [18], while the concentration of VFA and its individual molar proportions were determined using a gas chromatograph (GC, Thermo fisher scientific, Inc., TRACE1300, Rodano, Milan, Italy). The GC was fitted with an AS3800 autosampler and equipped with a capillary column HP-FFAP (19091F-112; 0.320 mm o.d., 0.50 μm i.d., and 25 m length; J & W Agilent Technologies Inc., Palo Alto, CA, USA). A mixture of known concentrations of individual short-chain fatty acids was used as an external standard (Sigma Chemie GmbH, Steinheim, Germany) to calibrate the integrator.

### 2.6. Sampling and Analysis of Blood Serum

Blood samples were collected from all ewes on the last day of each month. Approximately 10 mL of blood was collected at 4 h postfeeding from the jugular vein into clean, dry tubes without anticoagulants. All blood samples were centrifuged at 4000× *g* for 20 min at 4 °C, and the serum was decanted into 2-mL Eppendorf tubes and frozen at −20 °C. Samples were analyzed for total proteins, albumin, urea-N, glucose, glutamate-pyruvate transaminase (GPT), glutamate-oxaloacetate transaminase (GOT), triglycerides, high-density lipoprotein cholesterol (HDL), low-density lipoprotein cholesterol (LDL), antioxidant capacity, beta-hydroxybutyrate (BHBA), and nonesterified fatty acids (NEFA) using test-specific kits (Stanbio Laboratory, Boerne, TX, USA) according to manufacturer instructions. Globulin concentration was calculated by difference (total protein–albumin).

### 2.7. Milk Sampling and Composition

During the sample collection period (the last 10 d of each month), individual ewes were hand-milked at 09:00 and 21:00 h daily, and 100 g/kg of recorded milk yield samples was taken at each milking and composited daily. Using infrared spectrophotometry (Lactostar Dairy Analyzer, Funke Gerber, Berlin, Germany), samples of milk were analyzed for individual component contents.

Fatty acid concentrations in milk were determined using methyl esters prepared by base-catalyzed methanolysis of the glycerides (potassium hydroxide in methanol) according to International Standards (International Standard ISO 15884-IDF 182. 2002. Brussels, Belgium: International Dairy Federation). An Agilent Technologies chromatograph (model 7890B, Santa Clara, CA, USA) equipped with a Zebron ZB-FAME column (60 m × 0.25 mm internal diameter × 0.25 μm film thickness) and a flame ionization detector (Perkin Elmer, Beaconsfield, UK) was used for the analysis of fatty acid concentrations. Analyses were carried out using hydrogen as the carrier gas at a flow rate of 1.8 mL/min, injection volume of 1 µL at a split ratio of 1:50 with the following temperature program: 100 °C for 3 min, rising at 2.5 °C/min to 240 °C and held for 10 min. The injector and detector (FID) temperatures were held at 250 °C and 285 °C, respectively. A mixture of known concentrations of individual fatty acids was used as an external standard (Supelco 37 Component FAME Mix certified reference material, TraceCERT^®^, in dichloromethane, Merck KGaA, Darmstadt, Germany) to calibrate the integrator.

The atherogenic index (AI) was calculated according to Ulbricht and Southgate [22].

The average yield (g/day) of each milk component was calculated by multiplying the milk yield by the component content (g/kg). The gross energy content in milk was calculated according to Tyrrell and Reid [23]. Fat-corrected milk (FCM, kg/day) and energy-corrected milk (ECM, kg/day) were calculated according to Tyrrell and Reid [23]. Feed efficiency was calculated and expressed as milk yield, FCM, and ECM per unit of DM intake.

### 2.8. Statistical Analyses

Data on the measured parameters during the experiment were analyzed in a completely randomized design with repeated measurements in time, where each ewe was an experimental unit using PROC MIXED of SAS (Online Version, SAS^®^ OnDemand for Academics, SAS Inst., Inc., Cary, NC, USA); the statistical model included the random effect of ewe (A_j_) with period (P_k_) and treatment (T_i_) as fixed effects using the following standard model:

Y_ijk_ = μ + T_i_+ A_j_(T_i_) + P_k_ + (T × P)_ik_ + E_ijk_

where Y_ijk_ expresses each observation of the *j*th ewe in the *k*th sampling time given *i*th treatment, T_i_ expresses the treatment effect, A_j_(T)_i_ expresses the ewe within diets, P_k_ expresses the sampling week effect, (T × P)_ik_ expresses the interaction between the diets and sampling period, and E_ijk_ expresses the experimental error. Period as a repeated measure with animal within treatment as the subject was considered in the model. The treatment sum of the square was partitioned into a single degree of freedom comparison to examine the linear and quadratic effect (orthogonal polynomial contrast) of each organic acid-treated DPL level. Contrast was also applied to test the difference between overall lactic acid- versus malic acid-treated DPL. The period and diet × period interactions were nonsignificant (i.e., *p* > 0.05) for most of the measurements; thus, only the main effects of diets were reported. Significance was declared at a level of *p* < 0.05.

## 3. Results

### 3.1. Feed Intake and Nutrient Apparent Digestibility

Lactic and malic acid-treated DPL silage increased (linear and quadratic effects, *p* < 0.01) DPL and total feed intakes, with no differences between malic and lactic acid (Table 2).

Increased (linear and quadratic effects, *p* < 0.01) DM, OM, CP, EE, and NSC digestibilities were observed with malic and lactic acid treatments (Table 2). Lactic acid treatment showed increased (*p* = 0.028) DM digestibility compared with malic acid treatment. The digestibility of NDF, ADF, cellulose, or hemicellulose was unaffected by organic treatments.

### 3.2. Ruminal Fermentation

The treatments did not affect ruminal butyrate concentration and the acetate: propionate ratio (Table 3). Ruminal NH_3_-N, total VFA, acetate, and propionate increased in a linear and quadratic way with increasing levels of malic and lactic acid-treated DPL silage; however, ruminal pH decreased, with no differences between the two organic acid treatments.

### 3.3. Blood Chemistry

Concentrations of total proteins, albumin, globulin, GOT, urea-N, GPT, triglycerides, HDL, LDL, NEFA, and BHBA were not affected by organic acid treatments, but malic and lactic acids increased (linear and quadratic effects, *p* < 0.01) serum glucose concentration and total antioxidant capacity (Table 3). Lactic acid treatment showed higher (*p* = 0.001) serum glucose concentration compared with the malic acid treatment.

### 3.4. Milk Yield, Composition, and Fatty Acids

The treatments did not affect daily milk production (Table 4); however, lactic (linear and quadratic effects, *p* < 0.05) and malic (linear effects, *p* < 0.01) acid treatments increased the daily production of ECM, FCM, and milk energy output, and the daily production of milk components. Increased contents of milk fats were observed with malic and lactic acid (linear and quadratic effects, *p* < 0.001) without affecting other milk components. Moreover, lactic and acid treatments linearly increased (*p* = 0.001) feed efficiency calculated as ECM: intake and FCM: intake without affecting feed efficiency calculated as milk: intake.

The treatments affected the proportions of most individual milk fatty acids (Table 5). With no differences between them (except for C14:0, C14.1, C15:0, C16:0, C16:1, C17:0, and C18.1n9 *trans*), lactic and malic acids treatments increased (linear and quadratic effects, *p* < 0.05) the proportions of total unsaturated fatty acids (UFA), polyunsaturated fatty acids (PUFA), total CLA, as well as the UFA: saturated fatty acids (SFA) ratio, and decreased the atherogenicity index.

## 4. Discussion

As previously noted [12], ensiling DPL with malic or lactic acids usually did not affect their major nutrient composition except for decreased NDF content. The decreased fiber content in malic and lactic acid-treated DPL may be due to hydrolysis or solubilization of cell wall fractions by the action of organic acids during the ensiling process [17,24].

### 4.1. Feed Intake and Nutrient Digestibility

Ensiling with lactic and malic acids increased total intake, which may be related to improved ruminal fermentation and digestion in ewes. Increasing feed intake with greater nutrient digestibility may explain the observed improvement in milk production. Organic acids added to the total mixed ration of Holstein cows increased feed intake [24]; however, El-Zaiat et al. [10] observed a decrease in feed intake by 3.7% due to malic acid supplementation to lactating cows at 50 g per cow per day. Yang et al. [16] observed unaffected daily intake of steers on a diet containing corn grain steeped in tap water containing 1% lactic acid (*w*/*v*) for 48 h. The method of administration (e.g., administration during ensiling or directly fed to animals) may be the reason for this discrepancy, and it indicates that administrating organic acids during ensiling can overcome the problem of the low palatability of organic acids.

Lactic and malic acids increased DM, OM, CP, EE, and NSC digestibilities, which may be attributed to the stimulation of different microbial growths involved in OM degradation [25]. Additionally, Papatsiros et al. [26] stated that organic acids promote enzymatic activities, increase pancreatic secretions, and stimulate the growth of beneficial bacterial populations [26]. El-Zaiat et al. [10] observed increased DM and OM digestibilities with malic acid administration to lactating cows. Fumaric acid at 2% of the diet of sheep increased the activities of xylanases and amylase in the rumen [27]. Ebrahimi et al. [28] and Genç et al. [29] observed that malic acid administration decreased protozoal numbers in a dose-dependent manner. Decreasing protozoa increases ruminal bacteria because protozoa are the main predator of ruminal bacteria [30], which may explain the observed digestibilities.

Lactic acid treatment increased DM digestibility compared with malic acid, which may be related to the long-chain organic acids [31]. Genç et al. [29] stated that the effect of organic acids on ruminal bacteria depends on their chemical composition. Gram-positive bacteria are more sensitive to long-chain acids, while Gram-negative bacteria are more sensitive to acids with less than eight carbon atoms. The differences in carbon length may partially explain the difference in DM digestibility between them. Unexpectedly, organic acid treatments showed weak effects on fiber digestibility. However, organic acids have the ability to remove H_2_ from the rumen, resulting in increased fiber digestibility [32]. Similar to the present results, Ebrahimi et al. [28] observed that malic acid had no effect on the digestibility of fiber fractions in calves. Li et al. [31] reported that organic acids had no effects on ruminal *Fibrobacter succinogenes* and *S. ruminantium*, which are involved in fiber digestion [33].

### 4.2. Ruminal Fermentation

The lowered ruminal pH is likely to be related to the greater concentration of total VFA. Moreover, lower silage pH during ensiling may also be a reason for lowered ruminal pH with feeding DPL ensiled with lactic or malic acid. The values of ruminal pH in the organic acid treatments were not substantially lower than the control treatment to reduce pH-induced fiber digestion, with the optimum pH level for fiber-degrading ruminal microbial activities and growth being 6.2 [34]. This can partially explain the minimal effects of treatments on fiber digestion. Organic acids can enhance lactate-utilizing bacterial activity and, thus, reduce lactate concentration in the rumen [35]. A decrease in ruminal lactate concentration is more favorable to increasing ruminal pH values, which may equal the acidity in the rumen raised by feeding low-pH materials [16]. Vyas et al. [36] observed unaffected ruminal pH with organic acids feeding to steers. However, steeping corn grains in water containing 1% lactic acid increased ruminal pH in steers [16], and fumaric acid feeding to sheep at 2% of the diet increased ruminal pH [27].

The increased concentration of ruminal NH_3_-N with organic acid administration may be a result of increased CP digestibility. Increasing the concentrations of ruminal NH_3_-N and VFA at the same time encourages the synchronization of dietary protein and energy, which is likely to enhance microbial-N production in the rumen [37]. Organic acids increased the concentrations of total VFA, indicating improved fermentation efficiency due to the buffering properties of organic acids [25]. The increased feed intake and improved OM and NSC digestibilities may increase TVFA concentrations in the rumen. Abdelrahman et al. [15] observed that feeding malic acid to lambs increased the production of ruminal VFA and propionate. Supplementation of fumarate at 2% of the diet increased total VFA concentration in the rumen of sheep [27]. In their review, Sahoo and Jena [25] stated that malic acid increases ruminal total VFA, propionate, and acetate concentration.

In contrast, organic acid treatments did not increase fiber digestibility; they increased acetate and propionate concentration in the present study. Carro and Ranilla [38] observed that malic acid increased both the ruminal propionate and acetate concentration at the same time because malic acid can be converted to acetate and propionate using different pathways, unlike other additives that can increase propionate at the expense of acetate. This may be partially explained based on the ability of organic acids to increase the activity of lactate-utilizing bacteria and the reduction in lactate concentration. It is known that propionate is derived from lactate through the reductive route, and the utilization of acetate is necessary as a cofactor for the conversion of lactate to propionate [39]. In the present experiment, fermentation measurements were conducted 3 h postfeeding, which may explain the increased acetate and propionate at the same time. Organic acids have the ability to stimulate the growth of *S. ruminantium*, which utilize lactate as a source of energy, resulting in increased propionate production [25]. Yang et al. [16] observed that steeping corn grain in water containing 1% lactic acid decreased acetate and did not affect propionate concentrations.

### 4.3. Blood Chemistry

The presence of all measured parameters in blood serum within the reference ranges indicates the good health, nutritional, physiological, and pathological status of ewes. The lack of effect of organic acid treatments on serum total proteins, albumin, globulin, and urea-N suggests the unaltered nutritional status of the ewes, protein catabolism, and normal kidney function in the ewes [40]. Additionally, there were no effects on serum GPT and GOT, indicating normal liver health [41]. The treatments did not affect the concentrations of triglycerides, HDL, NEFA, BHBA, and ADL, indicating that the treatments had no effects on liver dysfunction, fat metabolism, and mobilization of body fat [42]. Organic acid treatments increased serum glucose concentration, which was also observed in other experiments [10,43] as a result of improved OM and NSC digestibility as well as increased ruminal propionate. The increased serum total antioxidant capacity with malic and lactic acids confirms the results previously noted by Sharifi et al. [44], who observed that feeding date palms to lactating goats improved total antioxidant capacity in milk and blood. The presence of antioxidants and phenolic compounds in DPL may explain the observed results of serum total antioxidant capacity [44].

### 4.4. Milk Yield, Composition, and Fatty Acids

The treatments had no effects on daily milk production but increased daily ECM and FCM as well as feed efficiency (ECM: intake or FCM: intake ratios), indicating improved energy efficiency with the feeding of organic acid-treated DPL. The cumulative effects of the increased feed intake, nutrient digestibility, ruminal propionate, and serum glucose can explain these results. As previously noted, ruminal propionate is the precursor for lactose synthesis, and an increased supply of glucogenic precursors can increase milk yield [45]. Moreover, increased blood glucose indicates a better energy status in animals, which may also contribute to increased milk production [45]. El-Zaiat et al. [10] observed increased daily milk production by cows fed organic acids. Different organic acids added to the total mixed ration also increased FCM milk production in Holstein cows [24].

The treatments increased the fat contents in milk. Fat concentration in milk is related to diet composition and metabolic processes in the rumen, and this variable is often used as an indicator of rumen health and fiber adequacy in dairy animals [46,47]. The increased milk fat content may be related to increased ruminal acetate concentration, as acetate is the main precursor of fatty acid synthesis in the mammary gland [47]. Iqbal et al. [48] observed that feeding lactating cows a diet containing barley grain steeped in lactic acid increased milk fat content without affecting milk production and other variables of milk.

No effects of organic acids were found on most of the milk fatty acids. The treatments increased the proportions of C18.1n9 *trans*, C18:2 *trans*-10, *cis*-12, C18:2 *cis*-9, *trans*-11, C18.3n3, and C18.3n6. Nutritional factors account for approximately half of the variation in milk fat composition [49]. Around 60% of milk fatty acids come from plasma uptake, whereas the remaining amounts are synthesized in the mammary gland [50]. The observed alterations in ruminal fermentation and expected changes in the ruminal microbial population and biohydrogenation may be associated with altered milk fatty acid profiles [47].

Organic acids increased the proportions of PUFA and total CLA and lowered the atherogenicity index, indicating an improved nutritive value of produced milk. The increased PUFA concentrations are a result of increasing their absorption from the small intestine [51] as a result of escaping ruminal biohydrogenation, which makes them available for incorporation into milk fat [52]. Milk CLA content can arise as a result of the partial biohydrogenation of linoleic acid by ruminal bacteria (the primary source) or from de novo synthesis in the mammary gland (the secondary source) [53]. The concentration of CLA in milk is mainly related to the diet, which can result in up to a 10-fold increase in the concentration of CLA in milk [54]. The reason for these results is not clearly known in the present study. Malic acid and citric acid as silage additives increased C18:3n3 and PUFA content of alfalfa silages, probably due to reduced lipoperoxidase activity at low silage pH [55]. Similarly, malic acid and lactic acid treatment of DPL silage may increase the contents of PUFA and other UFA due to the low pH for organic acid-treated silages in the present study. Silage fermentation increases the availability of phenolic and saponin compounds, and organic acid-treated DPL may contain high concentrations of available phytochemical compounds. Different phytochemicals can decrease or modify ruminal biohydrogenation of PUFA, which subsequently may be absorbed into the blood, increasing PUFA, partially biohydrogentaed UFA, and CLA content in milk [56]. To our knowledge, no experiments have evaluated the effects of organic acids feeding to ruminants on milk fatty acid composition to compare the present results.

## 5. Conclusions

Date palm leaves can be used as a feed for lactating ewes under desert conditions. Feeding lactic or malic acid-treated DPL to lactating ewes at 20 or 40% of the total diet increased feed intake, improved nutrient digestibility, ruminal fermentation, and daily production of ECM and FCM, as well as feed efficiency. No major differences were observed between malic and lactic acid treatments, and, therefore, both of them are recommended at 5% during ensiling of DPL as a feed for lactating ewes.

## Figures and Tables

**Table 1:** Chemical composition of ingredients and diets (g/kg DM) fed to lactating ewes.

Item	Ingredient ^1^				Diet ^2^				
	CFM ^3^	DPL-No Additives ^4^	DPL-Lactic Acid ^5^	DPL-Malic Acid ^6^	Control	LAC50	LAC100	MAL50	MAL100
DM (g/kg fresh weight)	903	741	740	744	838	838	838	839	840
OM	923	907	909	911	917	917	917	917	918
CP	165	47	52	48	118	119	120	118	118
EE	47	22	19	22	37	36	36	37	37
NSC	414	276	302	286	359	364	369	361	363
NDF	297	562	536	555	403	398	393	402	400
ADF	175	316	300	316	231	228	225	231	231
Cellulose	142	194	179	197	163	68	68	68	67
Hemicellulose	122	246	236	239	172	160	157	163	164

ADF, acid detergent fiber; CP, crude protein; DM, dry matter; EE, ether extract; NDF, neutral detergent fiber; NSC, nonstructural carbohydrates; OM, organic matter; SEM, standard error of the mean. ^1^ Ingredients: CFM, concentrate feed mixture; DPL-no additives, date palm leaves ensiled without additives; DPL-lactic acid, date palm leaves ensiled with lactic acid; DPL-malic acid, date palm leaves ensiled with malic acid. ^2^ Diet: Control diet contained 600 g of concentrate feed mixture and 400 g of date palm leaves ensiled without additives, or the DPL ensiled without organic acids was replaced with DPL ensiled with lactic acid (LAC50 and LAC100, respectively) or malic acid (MAL50 and MAL100, respectively) at 50 or 100%. ^3^ The concentrate feed mixture consisted of (per kg DM): 540 g corn, 250 g wheat bran, 180 g soybean meal, 20 g limestone, 5 g sodium chloride, and 5 g minerals and vitamins mixture (containing per kg: 141 g Ca, 87 g P, 45 g Mg, 14 g S, 120 g Na, 6 g K, 944 mg Fe, 1613 mg Zn, 484 mg Cu, 1748 mg Mn, 58 mg I, 51 mg Co, 13 mg Se, 248,000 IU vitamin A, 74,000 IU vitamin D3, 1656 IU vitamin E). ^4^ pH = 4.3, ammonia-N = 64 g/kg of total N, volatile fatty acids = 73.0 g/kg DM, aflatoxin F_1_ = 3.2 µg/kg DM. ^5^ pH = 3.8, ammonia-N = 49 g/kg of total N, volatile fatty acids = 93.0 g/kg DM, aflatoxin F_1_ = 3.3 µg/kg DM. ^6^ pH = 3.7, ammonia-N = 47 g/kg of total N, volatile fatty acids = 92.0 g/kg DM, aflatoxin F_1_ = 3.2 µg/kg DM.

**Table 2:** Intake and nutrient digestibility of diet containing date palm leaves ensiled without additives or ensiled with lactic acid or malic acid and fed to lactating Farafra ewes ^1^.

Item		Lactic Acid		Malic Acid		SEM	*P*-Lactic Acid		*P*-Malic Acid		*P*-Lactic vs. Malic
	Control	LAC50	LAC100	MAL50	MAL100		Linear	Quadratic	Linear	Quadratic	
Intake (g DM/day) ^2^											
DPL	309	329	332	334	334	2.2	<0.001	0.003	<0.001	<0.001	0.152
Total	819	839	842	844	844	2.2	<0.001	0.003	<0.001	<0.001	0.152
Digestibility (g absorbed/kg ingested)											
DM	561	625	620	609	611	5.4	<0.001	<0.001	<0.001	0.001	0.028
OM	562	628	628	621	633	6.4	<0.001	0.001	<0.001	0.004	0.152
CP	553	626	625	619	621	4.3	<0.001	<0.001	<0.001	<0.001	0.225
EE	581	619	622	623	627	4.6	<0.001	0.003	<0.001	0.001	0.363
NSC	557	607	600	604	611	5.5	<0.001	<0.001	<0.001	0.004	0.415
NDF	520	545	547	535	535	6.1	0.111	0.061	0.098	0.153	0.069
ADF	515	545	543	537	539	5.4	0.164	0.441	0.082	0.117	0.103
Cellulose	528	562	558	562	561	6.8	0.091	0.074	0.084	0.761	0.521
Hemicellulose	524	564	556	559	560	7.9	0.213	0.079	0.165	0.098	0.874

ADF, acid detergent fiber; CP, crude protein; DPL, date palm leaves; DM, dry matter; EE, ether extract; NDF, neutral detergent fiber; NSC, nonstructural carbohydrates; OM, organic matter; SEM, standard error of the mean. ^1^ Diet: Control diet contained 600 g of concentrate feed mixture and 400 g of date palm leaves ensiled without additives, or the DPL ensiled without organic acids was replaced with DPL ensiled with lactic acid (LAC50 and LAC100, respectively) or malic acid (MAL50 and MAL100, respectively) at 50 or 100%. ^2^ All ewes were fed the same amounts of concentrates (510 g DM/ewe/day).

**Table 3:** Ruminal fermentation and blood measurements of lactating Farafra ewes fed a diet containing date palm leaves ensiled without additives or ensiled with lactic acid or malic acid ^1^.

Item		Lactic Acid		Malic Acid		SEM	*P*-Lactic Acid		*P*-Malic Acid		*P*-Lactic vs. Malic
	Control	LAC50	LAC100	MAL50	MAL100		Linear	Quadratic	Linear	Quadratic	
Ruminal fermentation											
pH	5.71	5.39	5.39	5.38	5.38	0.046	<0.001	0.005	<0.001	0.005	0.846
Ammonia-N, mg/L	301	331	333	331	327	3.8	<0.001	0.002	<0.001	0.001	0.410
Total VFA, mmol/L	121	134	134	132	132	1.2	<0.001	<0.001	<0.001	0.001	0.121
Acetate, mmol/L	71.9	80.7	82.0	79.2	80.0	0.914	<0.001	0.001	<0.001	0.004	0.054
Propionate, mmol/L	27.2	29.7	31.0	30.7	30.6	0.47	<0.001	0.301	<0.001	0.002	0.494
Butyrate, mmol/L	21.6	23.5	21.2	22.2	21.5	0.69	0.659	0.112	0.888	0.392	0.475
Acetate: propionate ratio	2.67	2.74	2.66	2.60	2.63	0.048	0.934	0.222	0.561	0.374	0.070
Blood chemistry											
Total proteins, g/dL	7.23	7.60	7.60	7.57	7.61	0.135	0.061	0.065	0.092	0.070	0.754
Albumin, g/dL	3.86	4.05	4.05	4.05	4.07	0.098	0.152	0.062	0.089	0.082	0.763
Globulin, g/dL	3.37	3.55	3.55	3.52	3.54	0.095	0.056	0.107	0.088	0.236	0.665
Urea-N, mg/dL	23.5	23.6	23.6	23.4	23.3	0.15	0.576	0.832	0.679	0.987	0.242
Glucose, mg/dL	61.7	68.2	68.4	67.4	67.9	0.18	<0.001	<0.001	<0.001	<0.001	0.001
GPT, Units/L	15.6	15.5	16.0	15.4	15.6	0.33	0.109	0.069	0.800	0.222	0.117
GOT, Units/L	32.8	31.8	31.8	31.8	32.0	1.24	0.059	0.123	0.307	0.545	0.619
Triglycerides, mg/dL	164	170	171	171	170	4.56	0.083	0.192	0.120	0.069	0.932
HDL, mg/dL	93.6	94.9	94.5	94.2	95.1	0.43	0.133	0.119	0.114	0.783	0.921
LDL, mg/dL	71.0	71.1	71.3	71.8	71.0	0.50	0.684	0.991	1.000	0.201	0.720
Antioxidant capacity, mg/dL	99	110	110	110	111	1.72	<0.001	0.013	<0.001	0.012	0.750
BHBA, mg/dL	0.84	0.90	0.86	0.89	0.90	0.017	0.299	0.100	0.080	0.304	0.497
NEFA, mg/dL	1.73	1.77	1.77	1.76	1.76	0.054	0.599	0.840	0.693	0.859	0.877

BHB, beta-hydroxybutyrate; GOT, glutamate-oxaloacetate transaminase; GPT, glutamate-pyruvate transaminase; HDL, high-density lipoprotein cholesterol; LDL, low-density lipoprotein cholesterol; NEFA, nonesterified fatty acids; SEM, standard error of the mean; VFA, volatile fatty acids. ^1^ Diet: Control diet contained 600 g of concentrate feed mixture and 400 g of date palm leaves ensiled without additives, or the DPL ensiled without organic acids was replaced with DPL ensiled with lactic acid (LAC50 and LAC100, respectively) or malic acid (MAL50 and MAL100, respectively) at 50 or 100%.

**Table 4:** Milk production, composition, and feed efficiency of lactating Farafra ewes fed a diet containing date palm leaves (DPL) ensiled without additives or ensiled with lactic acid or malic acid ^1^.

Item		Lactic Acid		Malic Acid		SEM	*P*-Lactic Acid		*P*-Malic Acid		*P*-Lactic vs. Malic
	Control	LAC50	LAC100	MAL50	MAL100		Linear	Quadratic	Linear	Quadratic	
Production, g/d											
Milk	603	642	644	641	662	14.7	0.053	0.186	0.071	0.549	0.498
Energy-corrected milk (ECM)	637	713	720	702	728	12.9	<0.001	0.029	<0.001	0.212	0.917
Fat-corrected milk (4% FCM)	617	688	696	681	705	12.4	<0.001	0.041	<0.001	0.191	0.953
Milk energy output, MJ/d	1.96	2.20	2.23	2.17	2.25	0.040	<0.001	0.025	<0.001	0.205	0.864
Total solids	80.2	89.4	90.1	88.0	91.3	1.61	<0.001	0.032	<0.001	0.253	0.958
Solids nonfat	55.1	60.7	60.9	59.7	62.0	1.11	0.001	0.049	<0.001	0.392	0.945
Fat	25.0	28.7	29.2	28.3	29.3	0.53	<0.001	0.015	<0.001	0.088	0.758
Protein	25.0	26.8	26.7	26.6	27.5	0.50	0.018	0.033	0.001	0.587	0.604
Lactose	25.5	28.8	29.0	28.1	29.2	0.53	<0.001	0.018	<0.001	0.292	0.612
Ash	4.61	5.08	5.13	5.09	5.28	0.116	0.002	0.151	<0.001	0.325	0.465
Composition, g/kg DM											
Total solids	133	139	140	138	138	2.4	0.661	0.081	0.091	0.111	0.082
Solids nonfat	91.5	94.5	94.5	93.3	93.7	2.28	0.157	0.081	0.136	0.077	0.182
Fat	41.6	44.7	45.4	44.2	44.3	0.23	<0.001	<0.001	<0.001	<0.001	0.001
Protein	41.5	41.7	41.5	41.6	41.5	0.23	0.862	0.410	0.939	0.929	0.854
Lactose	42.3	44.8	45.1	43.8	44.2	1.17	0.061	0.088	0.515	0.777	0.841
Ash	7.65	7.91	7.98	7.92	7.99	0.212	0.068	0.481	0.031	0.471	0.924
Milk energy content, MJ/kg DM	3.25	3.43	3.45	3.39	3.40	0.191	0.616	0.089	0.112	0.183	0.045
Feed efficiency											
Milk: intake	0.74	0.77	0.76	0.76	0.79	0.014	0.170	0.394	0.318	0.893	0.639
ECM: intake	0.78	0.85	0.85	0.83	0.86	0.016	0.001	0.074	0.001	0.603	0.774
FCM: intake	0.76	0.82	0.83	0.81	0.84	0.015	0.001	0.120	0.001	0.568	0.870

^1^ Diet: Control diet contained 600 g of concentrate feed mixture and 400 g of date palm leaves ensiled without additives, or the DPL ensiled without organic acids was replaced with DPL ensiled with lactic acid (LAC50 and LAC100, respectively) or malic acid (MAL50 and MAL100, respectively) at 50 or 100%.

**Table 5:** Milk fatty acid profile (g/100 g fatty acids) of lactating Farafra ewes fed a diet containing date palm leaves (DPL) ensiled without additives or ensiled with lactic acid or malic acid ^1^.

Item		Lactic Acid		Malic Acid		SEM	*P*-Lactic Acid		*P*-Malic Acid		*P*-Lactic vs. Malic
	Control	LAC50	LAC100	MAL50	MAL100		Linear	Quadratic	Linear	Quadratic	
C4:0	2.82	3.01	2.96	2.97	2.88	0.063	0.306	0.908	0.560	0.166	0.213
C6:0	2.05	2.11	2.07	2.09	2.10	0.036	0.240	0.747	0.259	0.802	0.689
C8:0	2.27	2.32	2.30	2.34	2.34	0.016	0.007	0.426	0.002	0.586	0.782
C10:0	5.07	5.12	5.12	5.14	5.13	0.035	0.441	0.144	0.407	0.552	0.187
C11:0	0.88	0.90	0.90	0.89	0.89	0.014	0.544	0.529	0.039	0.660	0.150
C12:0	3.21	3.23	3.23	3.22	3.22	0.026	0.762	0.746	0.124	0.219	0.063
C14:0	9	9.0	9	9	9	0.06	0.034	0.662	0.857	0.617	0.003
C14:1	0.67	0.69	0.69	0.69	0.69	0.004	0.105	0.426	0.001	0.044	0.014
C15:0	0.54	0.55	0.56	0.54	0.55	0.005	0.742	0.684	0.001	0.913	0.001
C16:0	26	24	24	24	24	0.15	<0.001	0.001	<0.001	<0.001	0.004
C16:1	1.20	1.28	1.30	1.23	1.27	0.008	0.003	0.405	<0.001	<0.001	<0.001
C17:0	0.89	0.92	0.94	0.89	0.91	0.007	0.885	0.933	<0.001	0.001	<0.001
C18:0	16.5	16.2	16.3	16.1	16.3	0.09	0.072	0.031	0.027	0.854	0.324
C18:1n9 *cis*	24.4	25.3	25.3	25.4	26.0	0.14	<0.001	0.001	<0.001	0.001	0.068
C18:1n9 *trans*	2.42	2.86	2.86	2.86	2.85	0.026	<0.001	<0.001	<0.001	<0.001	0.038
C18:2 *trans*-10, *cis*-12	0.28	0.30	0.30	0.30	0.30	0.005	0.001	0.035	<0.001	0.011	0.119
C18:2 *cis*-9, *trans*-11	0.17	0.19	0.19	0.19	0.19	0.011	0.245	0.400	0.281	0.040	0.510
C18:3n3	0.16	0.18	0.18	0.18	0.18	0.003	0.001	0.026	0.001	0.013	0.750
C18:3n6	0.36	0.39	0.40	0.40	0.39	0.004	<0.001	0.001	<0.001	0.001	0.183
C20:0	0.68	0.64	0.64	0.63	0.63	0.006	<0.001	0.006	<0.001	0.001	0.259
C20:5n3	0.15	0.17	0.18	0.17	0.17	0.004	<0.001	0.216	0.001	0.001	0.964
C22:5n3	0.19	0.22	0.22	0.22	0.22	0.005	<0.001	0.145	0.001	0.001	0.431
SFA	70	68	68	68	68	0.1	<0.001	<0.001	<0.001	<0.001	0.516
UFA	30	31	31	31	32	0.1	<0.001	<0.001	<0.001	<0.001	0.482
MUFA	28.7	30.2	30.2	30.2	30.8	0.14	<0.001	<0.001	<0.001	<0.001	0.376
PUFA	1.32	1.46	1.46	1.47	1.45	0.014	<0.001	0.001	<0.001	<0.001	0.074
Total CLA	0.45	0.49	0.49	0.49	0.49	0.012	0.011	0.110	0.003	0.004	0.224
n-6: n-3	2.20	2.21	2.26	2.25	2.24	0.045	0.662	0.867	0.300	0.949	0.620
UFA: SFA	0.43	0.46	0.46	0.46	0.47	0.003	<0.001	<0.001	<0.001	<0.001	0.700
Atherogenicity index ^2^	2.20	2.03	2.04	2.04	1.98	0.015	<0.001	<0.001	<0.001	<0.001	0.287

CLA, conjugated linoleic acid (C18:2 *trans*-10, *cis*-12, and C18:2 *cis*-9, *trans*-11); MUFA, monounsaturated fatty acids; PUFA, polyunsaturated fatty acids; SEM, standard error of the mean; SFA, saturated fatty acids; UFA, unsaturated fatty acids. ^1^ Diet: Control diet contained 600 g of concentrate feed mixture and 400 g of date palm leaves ensiled without additives, or the DPL was replaced with DPL ensiled with lactic acid (LAC50 and LAC100, respectively) or malic acid (MAL50 and MAL100, respectively) at 50 or 100%. ^2^ Calculated according to Ulbricht and Southgate [22]: Atherogenicity index = (C12:0 + 4 × C14:0 + C16:0)/∑ UFA.

## Data Availability

The datasets generated during and/or analyzed during the current study are available from the corresponding author on reasonable request.
